# Methods to Evaluate Bacterial Motility and Its Role in Bacterial–Host Interactions

**DOI:** 10.3390/microorganisms10030563

**Published:** 2022-03-04

**Authors:** Victoria Palma, María Soledad Gutiérrez, Orlando Vargas, Raghuveer Parthasarathy, Paola Navarrete

**Affiliations:** 1 Laboratory of Microbiology and Probiotics, Institute of Nutrition and Food Technology (INTA), University of Chile, El Líbano 5524, Santiago 7830490, Chile; victoria.palma@ug.uchile.cl (V.P.); soleguti@uchile.cl (M.S.G.); orlandovargasblanco@gmail.com (O.V.); 2 Millennium Science Initiative Program, Milenium Nucleus in the Biology of the Intestinal Microbiota, National Agency for Research and Development (ANID), Moneda 1375, Santiago 8200000, Chile; 3 Institute of Molecular Biology, University of Oregon, Eugene, OR 97403, USA; raghu@uoregon.edu; 4 Department of Physics and Materials Science Institute, University of Oregon, Eugene, OR 97403, USA

**Keywords:** bacterial motility, motility methods, bacteria, flagella, bacterial–host interaction, microscopy

## Abstract

Bacterial motility is a widespread characteristic that can provide several advantages for the cell, allowing it to move towards more favorable conditions and enabling host-associated processes such as colonization. There are different bacterial motility types, and their expression is highly regulated by the environmental conditions. Because of this, methods for studying motility under realistic experimental conditions are required. A wide variety of approaches have been developed to study bacterial motility. Here, we present the most common techniques and recent advances and discuss their strengths as well as their limitations. We classify them as macroscopic or microscopic and highlight the advantages of three-dimensional imaging in microscopic approaches. Lastly, we discuss methods suited for studying motility in bacterial–host interactions, including the use of the zebrafish model.

## 1. Introduction

Motility is defined as the movement of cells by some form of self-propulsion [1]. Many bacterial cells are motile as it allows them, for example, to escape from unfavorable conditions and to exploit new resources or opportunities. Combined with chemotaxis, the ability to sense a chemical gradient and direct movement accordingly, it enables bacteria to pursue nutrients and to reach specific niches. In this sense, motility is also involved in the interaction between microorganisms and their host, specifically in colonization or infectious pathogenic processes. Indeed, non-motile mutants are either impaired or completely disabled to colonize and/or cause disease [2].

There are different types of motility, often classified as swimming, swarming, twitching, gliding, and sliding [3,4]. Swimming consists of movement in a liquid environment typically by using flagella, long, thin appendages attached to the cell [1]. Swarming is a coordinated movement of cells that are propelled by flagella through thin liquid films on surfaces and can involve cellular differentiation into a longer and hyper-flagellated phenotype [5]. Other structural molecules can be involved in bacterial movement such as twitching and gliding, both being active ways of moving over a surface. In twitching, type IV pili extend and attach to a solid surface, then retract to allow movement [6]. While twitching is described as intermittent and uneven, gliding is a more organized and smoother cell movement that comprises evolutionarily unrelated machineries which include the use of adhesins that attach to a substratum and either move across the cell or use surface proteins to perform a back-and-forth motion [4,7,8]. Sliding is a passive movement that, instead of requiring an appendage, occurs by bacteria’s surfactants (i.e., rhamnolipids) [3]. While dividing, cells are pushed outwards by the growing colony, and surfactants reduce the surface tension decreasing the friction between the surface and bacterial cells, accelerating their spreading [9]. Alternatively, sliding can be attributed to osmolytes (i.e., glycine betaine) secreted by bacteria that draw water to the media surface [9].

Other types of motion are possible [4,10,11,12,13,14]. *Spiroplasma* propagates kinks along its helical body to swim [10], while it is believed that cyanobacteria of the genus *Synechococcus* does so by propagating spicule-like surface extensions along the cell [11,12]. Another example is *Acinetobacter baumannii* 17978, whose type I pili confer surface motility modulated by light [13]. Moreover, some parasitic bacteria can induce actin polymerization to form a tail and move inside the host cell. These motility types and others are included in a recent re-classification based on the structure of the force-producing motor [14].

Different motility types are not mutually exclusive. It has been shown that besides swimming, swarming, and twitching, *Pseudomonas aeruginosa* can also display sliding motility [15], and a recent review discusses different forms of movement observed in *Staphylococci*, including gliding and sliding in *Staphylococcus aureus* [16]. Motility also shows great variability among species and even strains. For example, strains from different serovars of *Salmonella enterica* showed differences up to a factor of 2.7 in swimming speed [17].

Although motility can provide fitness advantages, it also has considerable drawbacks, such as high energetic and metabolic cost [18], and the presence of antigenic structures such as flagella [19]. These costs are a function of the biological context, and therefore realistic assessment of motility requires setting experimental conditions to be as close as possible to the actual environment of interest. We will discuss here common and recent methodological approaches that have been used to study bacterial motility and its role in bacteria–host interactions.

## 2. Macroscopic Techniques

We will distinguish between macroscopic and microscopic methods for studying bacterial motility. The former does not resolve the motions of individual bacteria but rather the spread of a population through some medium. Qualitatively, the link between macroscopic spreading and microscopic motility makes sense—a non-motile species, for example, will have little dispersal, and a vigorously moving species may travel far. Quantitatively, the relationship between macroscopic dispersal and the motility of individual cells is more subtle because the spread of a population is driven by growth (cell division) as well as motility. For example, a bacterium *Escherichia coli* that travels in fairly straight “runs” of a constant speed, *ν_bacteria_* that persist on average for time *τ* before the organism “tumbles” and randomizes its direction, executes a random walk through its three-dimensional world with an effective diffusion coefficient *D* proportional to the square of its speed [20,21].

(1) $$D=\frac{1}{3}\;v_{bacteria}{}^{2}\tau$$

If the bacteria are also growing exponentially with growth rate r, the population will spread with a velocity:

(2) $$v=2\sqrt{rD}$$

as Fisher, Kolmogorov, and others showed nearly a century ago [22,23]. For typical bacterial swimming speeds and growth rates, the macroscopic dispersal speed (perhaps millimeters per hour) will be one or two orders of magnitude lower than the speed of individual bacteria (perhaps tens of microns per second). Besides considering the expansion described by Fisher (Fisher waves), recent work on bacterial range expansion has taken into consideration phenomena such as intraspecific cooperativity [24] and chemotaxis [25].

**Figure 1:**
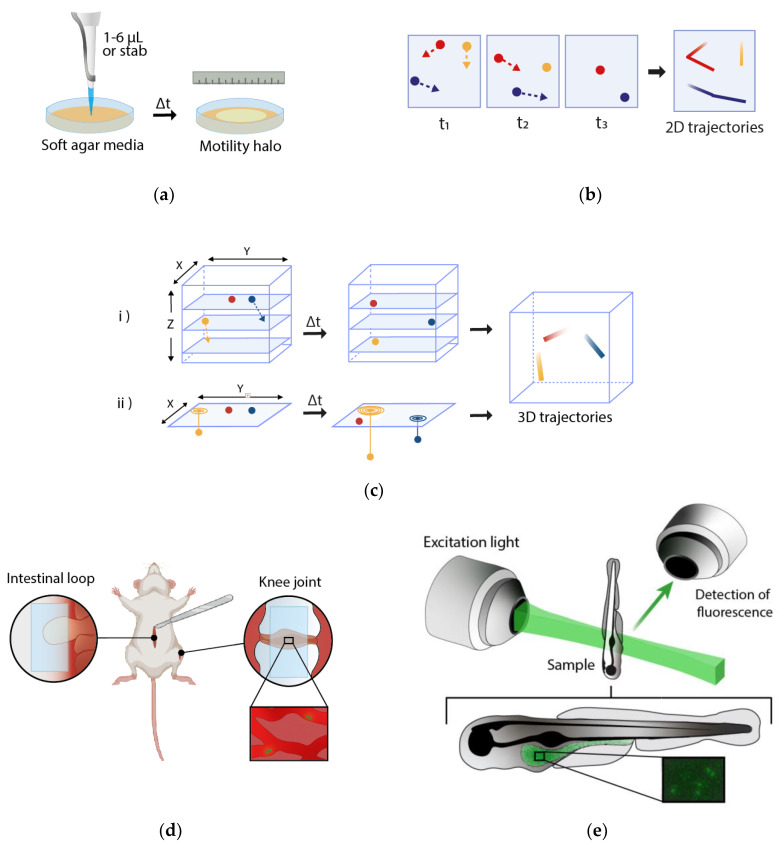
Some examples of methods to study bacterial motility and motility tracking. (**a**) Soft agar assay is the most common macroscopic method used to study motility. After inoculating 1–6 µL or a stab of bacterial culture in soft agar, motile bacteria will spread and blur the media. (**b**) Assessing motility using some common microscopic methods is based on tracking individual bacteria to obtain their 2D trajectories. If a cell leaves the focal plane (orange cell) the track ends. (**c**) Three-dimensional trajectories can be obtained by (**i**) stacks of 2D slices along the z-axis (z-stacking) or by (**ii**) projecting the 2D image in the z-axis according to certain parameters such as depth-dependent shape in the case of defocused imaging methods. (**d**) Intravital microscopy (IVM) aims to visualize phenomena occurring inside live animals. For example, exposing the tissue of an anesthetized mouse by doing small incisions while carefully preserving its physiological conditions, a glass coverslip can be placed in the knee joint [32] or an intestinal loop [33] to visualize the movement of fluorescently labeled bacteria. Bacteria (green) are shown inside knee joint microvasculature. (**e**) The optical transparency of zebrafish larvae allows non-invasive visualization of the in vivo motility of fluorescent bacteria with light-sheet fluorescence microscopy (LSFM) in which a focal plane is illuminated, exciting all points in the plane simultaneously.

The most common macroscopic approach to studying macroscopic motility is by examining bacterial spread through semi-solid agar (soft agar) [26]. Starting from an inoculation stab deep inside the agar, non-motile bacteria will remain near the inoculation zone, while motile bacteria will spread and visibly blur the media (Figure 1a). Because of its simplicity, it is particularly well suited to uncover non-motile or hypermotile strains (Table 1). Some bacteria can form, depending on the environmental conditions, characteristic colony patterns in plates, especially during swarming [5]. Spatial patterns seen using the soft agar method are linked to chemotaxis—directed motion induced by chemicals—as chemoattractants present in the agar that are metabolized by bacteria creating radial concentration gradients that boosts outward expansion [27]. Using low concentrations of the metabolizable chemoattractant would accentuate taxis response [28]. Other methods to study chemotaxis have been described, such as the capillary assay, where a capillary tube filled with a chemical is placed in a bacterial suspension and the accumulation of bacteria towards or away from the chemical is assessed visually [27,28].

In soft agar assays, the agar concentration can be adjusted according to the bacterial species and motility type (Table 2). To assess sliding motility, soft agar assays with flagellum- and/or type IV pili-deficient strains are usually used to discard swarming and/or twitching, respectively [9]. If the motility zone cannot be visualized because of low cell density, for example, in the case of using agar medium low in nutrients, the bacterial density of an agar plug at a standardized distance can be measured to determine if bacteria has reached this position [29]. Labeling can increase the contrast between the spreading bacteria and the culture media. For example, 2,3,5-triphenyltetrazolium chloride (TTC) can be easily incorporated into the media, coloring bacterial growth [30]. Genetically modified bacteria encoding fluorescent proteins (i.e., GFP) or bioluminescent bacteria can also be used. For example, a fluorescent *Pseudomonas* and a bioluminescent *Salmonella* can both be distinguished in a co-swarming experiment [31]. Staining the biosurfactant rhamnolipids produced by bacteria, by adding Red Nile in the medium, showed that its production on agar surfaces was associated with bacterial swarming motility [31].

Environmental factors can also affect motility in agar. Tremblay and Déziel [40] proved that incubation temperature, pH, and drying time of soft agar under laminar flow affected swarming. In fact, even the location of the plates within the laminar flow causes significant differences in the swarming speed. These factors can affect media wetness that causes differences in the thickness of the liquid layer. The wetter the surface, the easier it is for bacteria to overcome frictional forces and move. This makes the reproducibility of these methods difficult to achieve.

## 3. Microscopic Techniques

Direct observation of motile bacterial cells provides the clearest insights into their motility but is challenging due to the length and time scales involved, as well as the potential complexity of the microbe’s environments. Bacteria are typically around a micron in size, with speeds up to tens of microns per second for flagella-mediated swimming. Video capture rates of at least 10 frames per second (fps) are therefore needed if cellular positions in adjacent images are to be no more than a body-length apart, facilitating reconstruction of trajectories. Slower rates could capture transitions between straight runs and tumbles, but only rates of 10 fps or higher can capture information about instantaneous speed and angle changes [41]. Moreover, if the bacterial density is too high, bacteria will traverse each other constantly, making the reconstruction process difficult.

Even though bacteria can be tracked using simple bright-field imaging, its discerning from the background can be enhanced by techniques such as dark-field microscopy, differential interference contrast microscopy (DIC), and phase-contrast microscopy (Table 3). In dark-field microscopy, illumination comes from the side so that only light scattered by objects such as bacteria is detected, providing a bright signal on a dark background. This enables, for example, visualization of flagella in addition to bacterial cell bodies when using a high light intensity [1,42]. One-sided dark-field illumination variant is useful to simultaneously determine cell rotation and swimming speed in spirochetes [43]. In DIC microscopy and phase-contrast microscopy, the index of refraction gradients and phase shifts, respectively, are mapped onto intensity differences, enhancing the contrast of relatively transparent objects, making these methods suitable for assessing bacterial movement and orientation [44,45]. Recently, Smith et al. [46] were able to quantify twitching throughout a dense bacterial colony where individual cell tracking was not feasible using DIC microscopy. Substantially, the edge of the colony was observed by microscope and light changes over time were mapped and associated with areas with low and high motility within the field of view, where a higher modulation of light implies higher bacterial motility.

Fluorescent microscopy enables clear identification of labeled cells or even specific bacterial components such as flagella [47] (Table 3). Genetically encoded fluorescent proteins are routinely used in model bacterial strains, such as *E. coli* K12 or *P. aeruginosa* PAO1, and increasingly in non-conventional microbes, such as some *Aeromonas* and *Pleisomonas* isolates from the zebrafish intestinal microbiota [48]. Exogenous labels, such as fluorescent probes, can be simpler to apply but will be diluted as bacteria divide, and one must be aware that they can potentially alter bacterial function. Staining with DAPI, for example, halves the swimming speed of *Pseudomonas* species [49], and fusions of fluorescent proteins to components of the bacterial flagellar motor can alter its dynamics [50].

Microscopy in its forms mentioned so far provides views of a two-dimensional image. The truncated fragments of trajectories as bacteria move in and out of the focal plane still allow measurement of swimming speeds, durations of runs, and other characteristics (Figure 1b). Nonetheless, three-dimensional trajectories obtained through stacks of 2D slices (z-stacking) can be worthwhile, giving a more accurate characterization of motility patterns (Figure 1c). The main disadvantages are the requirement of rapid stack acquisition and the high amount of computational resources needed to process large stacks. On the other hand, methods based on 2D projection allow observing a larger volume in exchange for providing less exact measurements [51]. Berg’s classic identifications of *E. coli*’s runs and tumbles tracked a microbe in three dimensions through a feedback loop linking image intensities and stage positions [52]. This is a very precise approach but can only track a single cell.

More recent techniques allow three-dimensional imaging of many bacteria within a field of view. In defocused imaging methods, depth-dependent image shape allows localization along the axis perpendicular to the focal plane (“z”) (Figure 1c). This approach has long been used for non-bacterial imaging, e.g., nanoparticles [53], and has been applied to bacteria using fluorescence [54] as well as phase contrast [51] microscopy, with a z-range limit of 200 µm in the latter. Gray values can also be used to determine z-distance in cells close to the focal plane [55].

Another technique for three-dimensional reconstruction that has been applied to bacterial systems is digital holographic microscopy (DHM) [56] (Table 3). DHM reconstructs an image from the interference pattern produced by the specimen, illuminated by a coherent light source, although it does not support three-dimensional fluorescence imaging. While a low scattering efficiency of bacteria is a disadvantage, DHM has high imaging speed and, with recent improvements, a lateral resolution of less than 0.5 µm has been achieved [57,58]. Acres and Nadeau [59] described that DHM 2D projections generally suffice for calculating free-swimming bacteria speeds, but z-stacking is more accurate to study motility near a solid surface.

In light-field microscopy (LFM) a whole volume is illuminated and sampled in one snapshot, instead of using a bidimensional image as an input [60] (Table 3). Then, a microlens array translates depth information into a two-dimensional light field image, which can be computationally transformed back into a three-dimensional image. While LFM employs wide-field illumination, selective volume illumination microscopy (SVIM) is a variant that illuminates only the volume of interest, reducing the background noise and increasing the contrast, allowing a lateral resolution of 3 μm [61] (Table 3). Considering the high number of optimizations available, SVIM has a great potential for visualizing dynamic and complex interactions such as the bacterial flow of *Vibrio fischeri* within the seawater surrounding the light organ of its host, the Hawaiian bobtail squid (*Euprymna scolopes*), as well as the selective colonization of that organ by individual bacteria [61].

Differential dynamic microscopy (DDM) [62,63] relies on light scattering caused by a suspension of particles, instead of tracking (Table 3). The scattering forms a speckle pattern whose intensity will vary at a rate depending on the speed of the particles movement. These fluctuations lead to the differential intensity correlation function from which parameters such as speed and motile fraction can be extracted. While the great number of bacteria that can be processed simultaneously is a considerable advantage, this method is unsuited for obtaining more specific motility parameters. DDM is convenient to quickly evaluate motility responses at a whole-population level, such as the speed recovery after osmotic shocks of different magnitudes [64] and local speed changes caused by a light pattern projection in photokinetic *E. coli* genetically modified to swim smoothly with a light controllable speed [65].

All these techniques and more, under the appropriate conditions, are precise enough to reveal strategies for swimming, chemotaxis, and other behaviors. Lastly, new methods for extracting and assessing image-derived trajectories can be used to produce more accurate characterizations of the bacteria’s movement. Accordingly, Liang et al. [66] implemented an unsupervised cluster analysis to fractionate the swimming trajectories of *Azotobacter vinelandii* into run and tumble segments, and then extracted the motility parameters distribution for each segment by fitting mathematical distributions. Other examples are the algorithms developed by Vissers et al. [67] (available on GitLab) to determine the positions, and orientations of individual rod-shaped bacteria, and track and analyze their surface dynamics, discerning between adhering, diffusing, and swimming cells.

Several techniques are available to study the role that bacterial appendages play in motility. However, as they are not in the scope of this review, they will be only briefly presented. Common techniques for visualizing nanomachineries include electron microscopy (EM) and its variations: transmission EM, scanning EM, and cryo-EM [68] are used to observe and study the structure of these bacterial components. Specifically, cryo-EM has recently provided 3D structural models of motility- [4] and chemotaxis-related [69] components with high resolution. However, freezing the cell makes capturing the dynamics of the machinery unachievable. Recent advances in fluorescence microscopy have allowed studying the functionality of these bacterial components. The substitution of amino acid residues of flagellin for cysteines or pilin subunits and subsequent labeling them with maleimide fluorescent dyes has allowed the study of flagellar [70] and pili [68] dynamics in real time. Moreover, a label-free technique, interferometric scattering microscopy (iSCAT), has recently been used to study type IV pili motor dynamics three-dimensionally [71]. These advances are vastly improving our knowledge of how the molecular machinery of bacterial motility operates.

## 4. Study of Bacterial Motility in Bacterial–Host Interactions

The study of bacterial motility inside a host is a more complex affair, which is why many studies simulate host conditions in vitro. Soft agar can, up to some extent, mimic physical, chemical, and nutritional conditions inside and outside the host [29,72,73]. Furthermore, chambers can mimic environments such as xylem vessels [74], enabling the discovery that *Xylella fastidiosa* migrates against the flow via twitching motility, and anaerobiosis, allowing researchers to prove that *Clostridioides difficile* modulates its swimming speed in the presence of a metabolite related to its host colonization [75]. Likewise, vertical diffusion chambers (VDC) were used to study the role of motility in *Campylobacter jejuni* invasion of epithelial cells [76]. An alternative closer to in vivo conditions is tissue culture, which allows investigation of motility behavior in processes such as cell invasion and tumor colonization [77,78,79]. Lastly, artificial systems that reproduce the successive environmental niches of the human gastrointestinal tract can be used to simulate the host’s dynamic conditions [80]. A metagenomic analysis of a gastrointestinal model of the colon developed by The Netherlands Organization for Applied Scientific Research (TIM-2) inoculated with human gut microbes showed that higher iron availability resulted in an enrichment of motility and chemotaxis functions [81]. Meanwhile, an early ex vivo approach in infant mice includes the labeling of motile and non-motile strains of *Vibrio cholerae* with fluorescent antibodies to visualize and compare its distribution in the extracted infected tissue [82].

In vivo real-time imaging is crucial to understand the colonization dynamics of bacteria. Intravital microscopy (IVM) consists of imaging inside live animals and often relies on fluorescence microscopy (Figure 1d; Table 3). The main problem is the thickness of the tissue samples, as off-focus blur and light scattering limit the depth of imaging [83]. Confocal microscopy can suffice; Moriarty et al. [84] reported high-resolution multidimensional visualization of bacterial dissemination inside a living mammal using spinning disk confocal IVM, revealing that dissemination of *Borrelia burgdorferi* in microvasculature of mice is a multi-stage process. Nonetheless, the scattered fluorescence limits the imaging depth of confocal microscopy to tens of microns. On the contrary, with multiphoton fluorescence, which is based on the simultaneous absorption of two or more infrared or near-infrared photons, imaging can be deeper than 100 μm in tissue. This is possible because longer wavelengths can penetrate at higher depths, besides lowering endogenous autofluorescence. Moreover, as excitation occurs only in the focal plane, there is minimal bleaching in the rest of the tissue [85,86]. Because of its advantages, IVM has been widely applied to visualize bacterial motility in colonized organs, such as *B. burgdorferi* in the skin [86] and *V. cholerae* in the intestine [33].

Zebrafish (*Danio rerio*) is a particularly advantageous vertebrate animal model for studying host–bacterial interactions due to their optical transparency at the larval stage, allowing for non-invasive examination of bacterial movement inside a living vertebrate host (Figure 1e). There are considerable similarities between zebrafish and mammals [87]. The gut is anatomically organized in separate sections and the intestinal epithelium is constantly renewing its cells. There is a high degree of orthologue genes [88] and their regulation within the gut is similar. The immune system of teleost fish species shares several traits with the system of mammals including the presence of lymphoid tissues, cell-mediated responses, and mucosal immunity [89].

Another advantage of zebrafish is that larvae hatch at 2–3 days post-fertilization (dpf) and open their mouths at 3 dpf, facilitating the production of germ-free or axenic individuals, great tools to study bacterial–host interactions. Fluorescently labeled bacteria can be inoculated via immersion at this developmental stage and visualized both at a whole population and at a single-cell level [90,91]. Germ-free zebrafish larvae colonized with fluorescent bacteria proved to be useful to examine the relationship between bacterial motility and symbiosis within the intestine [92,93]. In the last few years, the use of the zebrafish model coupled with light-sheet fluorescence microscopy (LSFM, also known as selective plane illumination microscopy) has provided new insights into the field of bacterial dynamics within a living host [94,95,96,97,98]. In this technique, only the focal plane is illuminated, exciting all points in the plane simultaneously, while out-of-focus points are not excited, minimizing photodamage and photobleaching and increasing imaging speed compared to point scanning methods, while achieving much higher resolution than wild field microscopy [96,99]. These characteristics make LSFM very suitable to follow bacterial dynamics inside the whole intestine of zebrafish for several hours. Nevertheless, because of light diffraction, generating a thin plane of excitation light is difficult, causing a loss in resolution compared to confocal and multiphoton imaging.

Combining LSFM, larval zebrafish, and bacteria engineered with inducible switches for a flagellar motor component revealed that the swimming motility of a zebrafish-native *Vibrio* species was necessary for its persistence inside the host and avoidance of expulsion with intestinal flow [98]. In a separate study, live imaging revealed that sub-lethal doses of the broad-spectrum antibiotic ciprofloxacin promoted its bacterial aggregation and expulsion from the intestine [100].

Finally, transcriptomic approaches can be used to investigate the effect of host or environmental factors [101,102,103] and phenomena such as macrophage internalization [104] and host cell infection [105] in the transcriptional regulation of genes related to bacterial motility. Employing microarrays, Snyder et al. [106] first assessed an *E. coli* pathotype’s transcriptome in vivo from bacteria extracted from infected mice, showing that flagellar genes were downregulated compared to in vitro conditions. Interestingly, this transcriptome was performed from different urine samples taken across 10 days of the infection period. A similar experiment using an *E. coli* expressing a luminescent reporter for the flagellar gene *fliC* showed that its expression was upregulated during the pathogen’s ascension through the upper urinary tract, suggesting a major contribution of motility in the colonization of the urinary system [107]. Recently, a comparison by RNA-seq between *Pseudomonas plecoglossicida* infecting spleens of the fish *Larimichthys crocea* and those cultivated in vitro revealed an up-regulation of motility-related and flagellum-related genes during the fish infection [108].

It is important to consider that, as single-cell transcriptomic approaches are difficult to achieve in prokaryotes [109], only homogenized output from a population is usually obtained for bacteria, impeding the study of phenotypically distinct subpopulations that could be present in the sample. Recent works have focused on overcoming these difficulties with strategies including mRNA enrichment methods. Kuchina et al. (2021) modified SPLiT-seq—a technique that uses combinatorial indexing to label the eukaryotic RNA’s cellular origin—to optimize its performance in bacteria. This approach was able to assess the fraction of *Bacillus subtilis* PY79 population that expressed flagellin and surfactin while growing in a rich medium [110].

Lastly, proteomic approaches, particularly those based on mass spectrometry (MS)—which measures the mass-to-charge ratio of ionized molecules to identify them—have proven to be a notable tool for assessing abundance changes in bacterial proteins inside a host [111,112]. Proteomic studies using liquid chromatography MS showed that downregulation of *Salmonella enterica* Typhimurium proteins involved in virulence, chemotaxis, and flagellar systems occurs earlier in bacteria inside macrophages compared to bacteria internalized by epithelial cells, suggesting that different host cell types have a different impact on motility adaptations [112].

## 5. Discussion and Concluding Remarks

The crucial role of motility in bacterial survival, host colonization, and/or virulence is a fact. This mini review showed that multiple approaches are available to study motility, from soft agar to a wide variety of microscopic techniques. The optimal choice will depend on the specific questions or requirements of the experiment, such as the number of cells or strains to process, z range needed, and growth conditions. In host–bacterial interactions, in vitro set-ups can provide fair approximations to the host environment, whereas intravital microscopy allows in vivo tracking of bacteria within the host tissue. This approach benefits from techniques that allow a greater depth of imaging, namely, confocal, and multiphoton fluorescence microscopy. Alternatively, the zebrafish model allows direct visualization of bacteria inside the host. Assessing the expression level of motility-related genes is also feasible. All these approaches can be combined to have a wider outlook; for example, coupling semi-solid (soft) agar plates with microscopy visualization. Accordingly, Deforet et al. [55] observed that macroscopically, a *P. aeruginosa* hyperswarmer mutant spreads faster, yet does not swim faster than the wild-type at the single-cell level. Further investigation led to realize if this phenomenon is related to wider turns.

Overall, a considerable number of new methods and advances to study bacterial motility have emerged during the last decade, deepening our understanding of bacterial behavior. Nevertheless, there are several issues that still need improvement, such as protocol standardization in soft agar assays; facilitating the implementation of 3D tracking, mostly achieved by microscopy techniques that are technically demanding and/or require complex set-ups and extending the depth of imaging for bacteria within host tissue in in vivo motility studies.

## Figures and Tables

**Table 1:** Macroscopic assays to study bacterial motility.

Macroscopic Assay	Applications	References
Soft-agar tubes	Easily identification of motile and non-motile bacteria	[26]
Soft-agar plates	Quantification of motility level, and identification of a motility type (Table 2) or patterns at a population level	[5,9,26]
Using low concentrations of a metabolizable chemoattractant	Assessing chemotactic motility	[27,28]
Using fluorescent labelling	Identification of more than two bacteria in co-swarming experiments, increasing contrast with the media, and studying of motility-related compounds	[31]

**Table 2:** Agar concentration in media according to the type of motility type to assess in a semi-solid (soft) agar assay.

Motility Type	Agar Concentration	References
Swimming	~ 0.3%	[34]
Swarming (temperate)	0.5–0.8%	[35]
Swarming (robust)	>1.5%	[35]
Twitching ^1^	1%	[36]
Sliding	0.3–0.4%, or 1–2% has also been used	[37,38]
Gliding	≤7% in *Myxococcus xanthus*	[39]

^1^ The plate is inoculated at the bottom of the media instead of the top.

**Table 3:** Microscopic techniques to study bacterial motility and their main applications.

Microscopic Techniques	Advantages	Disadvantages	Applications
Bright field microscopy	Simplest, cheapest, and highly accessible	Resolution limited by the wavelength of light, low contrast	Rapidly identification of a motile bacteria
Dark field microscopy	Contrast enhancement of unstained samples	Resolution limited by the wavelength of light	Visualization of motile bacteria, flagella
Phase contrast microscopy	Contrast enhancement of unstained samples	Resolution limited by the wavelength of light	Visualization of motile bacteria, and bacterial orientation
Differential interference contrast microscopy (DIC)	Contrast enhancement of unstained samples, edges of the object are highlighted	Resolution limited by the wavelength of light	Visualization of motile bacteria, and bacterial orientation
Confocal microscopy or laser scanning confocal microscopy (LSCM)	High resolution imaging due to reduction of background fluorescence; to collect serial optical sections from thick samples. Contrast and definition are improved	May not be fast enough to capture relevant dynamics; limited to the number of excitation wavelengths available from common lasers; imaging depth limited	Visualization of motile bacteria in thin tissues
Spinning disk confocal microscopy	Image acquisition speed is higher than LSCM improving the observation of dynamic processes and reducing photodamage	Imaging depth limited; sensitive camera is needed	Visualization of motile bacteria in thin tissues
Multiphoton confocal microscopy	Deeper penetration in tissue (>100 μm) compared to LSCM	Higher phototoxicity and photobleaching in the focal plane compared to LSCM	Visualization of motile bacteria in thick living tissue
Light-sheet fluorescent microscopy (LSFM) or selective plane illumination microscopy (SPIM)	High 3D resolution images	Sample mounting may be challenging; reduced resolution in depth compared to confocal microscopy	Visualization of motile bacteria in thick living tissue
Light-field-based selective volume illumination microscopy (SVIM)	Captures a 3D volume in a single snapshot	Requires specialized hardware; smaller spatial range than SPIM	Visualization of motile bacteria in thick living tissue in a single snapshot
Digital holographic microscopy (DHM)	High imaging speed; high resolution; adjust focus after the image is recorded, since all focus planes are recorded simultaneously by the hologram	Low scattering efficiency of bacteria	Visualization of several free-swimming bacteria
Differential dynamic microscopy (DDM)	Great number of bacteria can be processed simultaneously	Unsuited for obtaining specific motility parameters	Quick evaluation of motility responses at a whole-population level
